# Different training durations and styles of tai chi for glucose control in patients with type 2 diabetes: a systematic review and meta-analysis of controlled trials

**DOI:** 10.1186/s12906-019-2475-y

**Published:** 2019-03-14

**Authors:** Ting-Wei Xia, Yue Yang, Wei-Hong Li, Zhao-Hui Tang, Zong-Run Li, Li-Jun Qiao

**Affiliations:** 1 Basic Medical College, Chengdu University of TCM, Chengdu, 610075 China; 2 School of Clinical Medicine, Chengdu University of TCM, Chengdu, 610075 China

**Keywords:** Tai chi, Taijiquan, Type 2 diabetes, Blood glucose, Systematic review, Meta-analysis

## Abstract

**Background:**

Physical activity is an important part of the diabetes management plan. However, the effects caused by different training durations and styles of Tai Chi have not been evaluated. We conducted an updated systematic review of the effects of Tai Chi on patients with type 2 diabetes based on different training durations and styles.

**Methods:**

We performed a search for Chinese and English studies in 8 databases. Two reviewers independently selected the eligible trials and conducted a critical appraisal of the methodological quality.

**Results:**

Seventeen trials were included. Tai Chi was found to have reduced fasting blood glucose (FBG) [SMD = − 0.54, 95% CI (− 0.91, − 0.16), *P* = 0.005] and HbA1c [SMD = − 0.68, 95% CI (− 1.17, − 0.19), *P* = 0.006] overall, compared with a control group. Considering the subgroup analysis, the pooled results showed that 24 movements or Yang-style Tai Chi did not significantly reduce FBG after a duration of ≤3 months [SMD = − 0.46, 95% CI (− 1.42, 0.50), *P* = 0.35] or > 3 months [SMD = − 0.50, 95% CI (− 1.49, 0.49), *P* = 0.32], nor did it reduce HbA1c [SMD = − 1.22, 95% CI (− 2.90, 0.47), *P* = 0.16] after a duration > 3 months in all studies. However, other styles of Tai Chi significantly reduced FBG [SMD = − 0.90, 95% CI (− 1.28, − 0.52), *P* < 0.00001] and HbA1c [SMD = − 0.90, 95% CI (− 1.28, − 0.52), P < 0.00001] after a duration > 3 months, while no significant reduction in FBG [SMD = − 0.34, 95% CI (− 0.76, 0.08), *P* = 0.12] or HbA1c [SMD = − 0.34, 95% CI (− 0.76, 0.08), P = 0.12] was found after a duration ≤3 months.

**Conclusions:**

Tai Chi seems to be effective in treating type 2 diabetes. Different training durations and styles result in variable effectiveness. The evidence was insufficient to support whether long-term Tai Chi training was more effective.

## Background

Lifestyle management is garnering more and more attention as a fundamental aspect of diabetes care and includes diabetes self-management education, physical activity, support (DSMES), medical nutrition therapy (MNT), and so on [1]. According to recent IDF statistics, there are approximately 451 million people (age 18–99 years) with diabetes in the world, and approximately 90% of all cases are type 2 diabetes. Meanwhile, an additional 374 million people with impaired glucose tolerance are at high risk of developing diabetes [2, 3]. Physical activity is an important part of the diabetes management plan, which plays a specific role in the prevention of diabetes complications and the management of blood glucose for those with type 2 diabetes [1].

Tai Chi, as a mind-body therapy, is recommended by the ADA for people with type 2 diabetes to increase flexibility, muscular strength, and balance [1]. Tai Chi is considered to be an enjoyable activity, combining meditation and gentle movements that involve the entire body, and it has been shown to have a high level of adherence [4]. The exercise intensity of Tai Chi mainly depends on its training style, posture, frequency and duration, which have caught the attention of researchers. Many studies have shown inconsistent results regarding the reduction of FBG and HbA1c for those with type 2 diabetes who practice Tai Chi. To date, five systematic reviews of Tai Chi relative to DM have been published [5–9].

To the best of our knowledge, most of the previous systematic reviews (SRs) have suggested that the evidence is insufficient to support Tai Chi as an effective therapy for type 2 diabetes. The latest SR [9], in which 14 studies were included, showed 11 studies with non-exercise and 7 studies with other aerobic exercises. Therefore, the number of included studies was inappropriate. Furthermore, the target heart rate was frequently used as a measurement of exercise intensity and could not be replaced by an exercise amount.

Therefore, it is necessary to produce an updated systematic review to comprehensively and systematically evaluate the effects of Tai Chi on glycaemic control in patients with type 2 diabetes based on different training durations and styles of Tai Chi.

## Methods

### Data sources and selection strategy

We performed a search for Chinese and English studies in the following databases: PubMed, Cochrane Central Register of Controlled Trials (CENTRAL), Web of Science, Ovid LWW, Chinese Biomedical Literature Database (CBM), China Knowledge Resource Integrated Database (CNKI), Wanfang Data, and China Science and Technology Journal Database (VIP). The search strategy included terms relating to or describing Tai Chi and type 2 diabetes. Studies published between when the database establishment and April 2018 were retrieved.

Bibliographies of the related published systematic reviews were also reviewed. An illustrative PubMed search strategy is as follows: “Type 2 Diabetes Mellitus, Noninsulin-Dependent Diabetes Mellitus, Ketosis-Resistant Diabetes Mellitus, Non Insulin Dependent Diabetes Mellitus, Non-Insulin-Dependent Diabetes Mellitus, Stable Diabetes Mellitus, Type II Diabetes Mellitus, NIDDM Diabetes Mellitus, Noninsulin Dependent Diabetes Mellitus, Maturity-Onset Diabetes Mellitus, Maturity Onset Diabetes Mellitus, MODY Diabetes Mellitus, Slow-Onset Diabetes Mellitus, Maturity-Onset Diabetes, Maturity Onset Diabetes, Type 2 Diabetes, Adult-Onset Diabetes Mellitus” and “Tai Ji, Tai-ji, Tai Chi, Chi, Tai, Tai Ji Quan, Ji Quan, Tai, Quan, Tai Ji, Taiji, Taijiquan, T’ai Chi, Tai Chi Chuan”.

### Inclusion criteria

Studies should meet the following inclusion criteria (PICO format): (1) Participants: no restrictions on patients’ age, gender, disease duration, case source, nationality, or race; with a clear diagnosis of type 2 diabetes; without any serious complications. (2) Intervention: participation in Tai Chi as the major intervention. (3) Control: any type of control group, including usual care or standard treatment, and any kind of exercise was acceptable. (4) Outcomes: primary outcomes were HbA1c and fasting blood glucose. Secondary outcomes were total cholesterol (TC), triglycerides (TG), high-density lipoprotein cholesterol (HDL-C), low-density lipoprotein cholesterol (LDL-C), and body mass index (BMI). (5) Study type: RCTs. Nonrandomized controlled trials (N-RCTs), as well as cohort studies and case-control studies, were excluded.

### Data selection

First, two independent investigators reviewed the titles and abstracts. Abstracts that did not meet the eligibility criteria were excluded, and those that did not provide sufficient information about the inclusion criteria were further reviewed. Next, the same investigators analysed the full texts, blinded to each other’s review. Differences between the reviewers were resolved by consensus.

### Data extraction

Two investigators independently performed the data extraction using pre-piloted, standardized forms. The collected data included: basic information; methodological characteristics; risk of bias (ROB); participants’ demographic details; interventions; outcomes, fall outs; results of outcomes; and other. All differences were resolved by consensus.

### Methodological quality of assessment

Two investigators independently assessed the methodological quality of the included studies using RevMan 5.3.0, according to the Cochrane Handbook criteria for judging the ROB with the “Risk of bias” assessment tool [10].

#### Statistical analysis

RevMan 5.3.0, provided by Cochrane Collaboration, was used to analyse the results of the studies. All outcomes were continuous variables, so we expressed them as the mean ± standard deviation and then calculated the mean difference (MD) and obtained the two-sided *P*-value and 95% confidence interval (CI). We used the complete case data as the analysis data.

As the meta-analysis of the primary outcomes showed significant heterogeneity, we performed separate subgroup analyses with different training durations and styles in the intervention groups, as well as a sensitivity analysis if necessary. Publication bias was estimated with a funnel plot.

## Results

### Literature screening

**Fig. 1:**
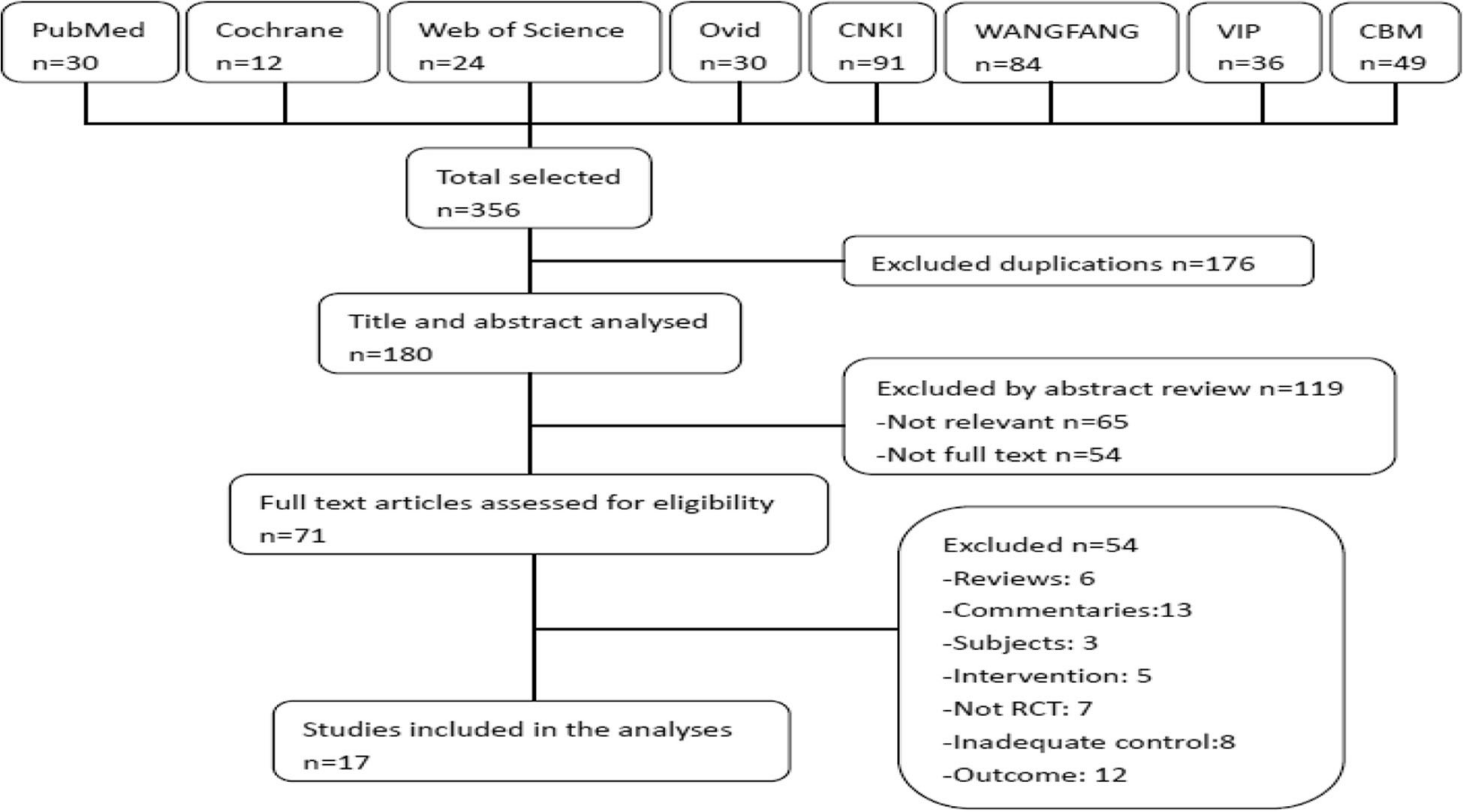
Flow chart of selection process

From the electronic bibliographic databases, we retrieved 356 original papers published between 1983 and 2018. After 176 duplicates were excluded, we screened the titles and abstracts of 180 publications; another 119 papers did not meet the inclusion criteria and were excluded. We downloaded the full text of the remaining 71 publications for further screening. We subsequently evaluated these retrieved articles by deep reading. Due to an ineligible study design, ineligible participants, inappropriate interventions, lack of controls, or unrelated outcome of primary interest, 54 studies were excluded. Finally, seventeen articles met the inclusion criteria completely and were analysed (Fig. 1).

### Characteristics of included studies

All seventeen trials were conducted in single centres (Table 1). The settings of the included studies were different, and included China [11–22], Taiwan (China) [23], Australia [24–26] and Thailand [27]. The simplified Tai Chi style was used in five RCTs [13, 14, 19, 20], Yang-style Tai Chi was used in two trials [15, 26], Chen-style was used in three trials [21, 22], Sun-style and Yang-style were used in two trials [24, 25], Lin-style was used in one trial [27], Da Yuan Jiang Tang-style was used in one study [16], Tai Chi Ball was used in two studies [12, 18], and two trials did not report the Tai Chi style [11, 17]. The interventions in the control group, which included standard diabetes care (diet control, injection of insulin or anti-diabetic medication, and usual care) [14, 16–19, 22, 27], active control (jogging, brisk walking, Yangko or social dancing for exercise, and other aerobic exercises) [13, 15, 21, 23, 25, 26], and no treatment (free activity programme, wait list) [11, 12, 24], could be regarded as conventional treatment or relaxation exercises. The sample sizes ranged from 16 to 100 participants. The number of Tai Chi sessions ranged from approximately 36–336. The number of supervised interventions ranged from two to seven sessions weekly, with 30–60 min per session.

**Table 1:** Characteristics of included studies

Authors, year	Location	Participants, Duration of disease (years)	No.	Tai Chi group			Control group	Follow-up	Outcome measures
				Form or style	Duration(min)	Frequency			
Zhang Y, 2008	China	Women with T2DM (4.4)	T: 10; C: 10	n.r.	60 min	Five times weekly	No treatment (free activity programme, n.r. in detail)	14 weeks	(1) FBG; (2) TC, HDL-C, LDL-C, TG
Chen SC, 2010	Taiwan (China)	Patients with T2DM (8.5/7.8)	T: 56; C: 48	Chen-style	60 min	Three times weekly	Conventional exercise (aerobic exercise plus home-based exercise, 60 min, 3 times weekly for 12 weeks)	12 weeks	(1) HbA1c, FBG
Lam P, 2008	Australia	Adults with T2DM (≥0.5)	T: 28; C: 25	Yang- and Sun-style 20-form	60 min	Two classes weekly	No treatment (wait list)	24 weeks	(1) HbA1c; (2) TC, TG
Youngwanichsetha S, 2013	Thailand	Women with T2DM (2.62)	TG:32; CG:32	18 movements (Lin’s style)	50 min	Three times weekly	Standard diabetes care (usual care, n.r. in detail)	12 weeks	(1) HbA1c, FBG; (2) BMI
Wang P, 2009	China	Older adults with T2DM (> 1)	TG:28; CG:26	Yang-style Tai Chi	30–50 min	Five or seven times weekly	Social dancing exercise (30–50 min every day for 24 weeks)	24 weeks	(1) HbA1c, FBG; (2) TC, HDL-C, LDL-C, TG
Zhao G, 2017	China	Patients with T2DM (n.r.)	T: 8; C: 8	Chen-style	60 min	Seven times weekly	Standard diabetes care (diet control and anti-diabetic medication, n.r. in detail)	16 weeks	(1) FBG; (2) TC, HDL-C, LDL-C, TG, BMI
Kan Y, 2004	China	Patients with T2DM (> 3)	T: 26; C: 22	24 movements (simplified version)	60 min	Seven times weekly	Walking or running (60 min every day for 12 weeks)	12 weeks	(1) FBG; (2) TC, HDL-C, LDL-C, TG, BMI
Wang JH, 2003	China	Older adults with T2DM (> 2)	T: 10; C: 6	24 movements (simplified version)	60 min	Seven times weekly	Standard diabetes care (usual medical care, n.r. in detail)	12 weeks	(1) FBG; (2) TC, TG, BMI
Yan J, 2004	China	Patients with T2DM (3.0/2.9)	T: 10; C: 8	Da Yuan Jiang Tang-style	30–60 min	Twice daily	Standard diabetes care (diet control and anti-diabetic medication, n.r. in detail)	24 weeks	(1) HbA1c, FBG
Li XB, 2013	China	Older adults with T2DM (3 months-10 years)	T: 30; C: 30	n.r.	45 min	Once daily	Standard diabetes care (injection of insulin or anti-diabetic medication, n.r. in detail)	8 weeks	(1) FBG
Wu F, 2010	China	Patients with T2DM (1.35/1.36)	T: 20; C: 20	24 movements (simplified version)	60 min	Three times weekly	Standard diabetes care (diet control and anti-diabetic medication, n.r. in detail)	24 weeks	(1) HbA1c, FBG
Wei DL, 2012	China	Patients with T2DM (0.5–3)	T: 26; C: 26	36 movements (Tai Chi Ball)	60 min	Once daily	Standard diabetes care (antidiabetic medication, n.r. in detail)	12 weeks	(1) HbA1c, FBG; (2) TC, HDL-C, LDL-C, TG, BMI
Li HC, 2015	China	Patients with T2DM (7.83/8.14)	T: 50; C: 50	Chen-style	40–50 min	Once daily	Sham exercise (jogging, brisk walking, Yangko and other aerobic exercises), 40–50 min for 6 months	24 weeks	(1) HbA1c, FBG; (2) TC, HDL-C, LDL-C, TG, BMI
TSANG T, 2008a	Australia	Adults with T2DM (8.7/12.4)	T: 18; C: 20	Yang-style	60 min	Twice weekly	Sham exercise (calisthenics and gentle stretching)	16 weeks	(1) HbA1c; (2) BMI
ORR R, 2006	Australia	Older adults with T2DM (8.5/9.0)	T: 17; C: 18	Sun-style and Yang-style	60 min	Twice weekly	Sham exercise (e.g., seated calisthenics, stretching)	16 weeks	(1) FBG
Xiao L, 2010	China	Patients with T2DM (1.35/1.36)	T: 12; C: 12	24 movements (simplified version)	60 min	Six times weekly	Standard diabetes care (anti-diabetic medication, glipizide, n.r. in detail)	24 weeks	(1) HbA1c, FBG
Xiao CM, 2015	China	Older adults with DM	T: 16; C: 16	Tai Chi Ball	1 to 2 h	Three sessions per week	No intervention	12 weeks	(1) HbA1c

Abbreviations: *T* Tai Chi, *C* control, *FBG* fasting blood glucose, *HbA1c* glycated haemoglobin, *TC* total cholesterol, *TG* triglycerides, *HDL-C* high-density lipoprotein cholesterol, *LDL-C* low-density lipoprotein cholesterol, *BMI* body mass index, *n.r* not reported

### Methodological quality

**Fig. 2:**
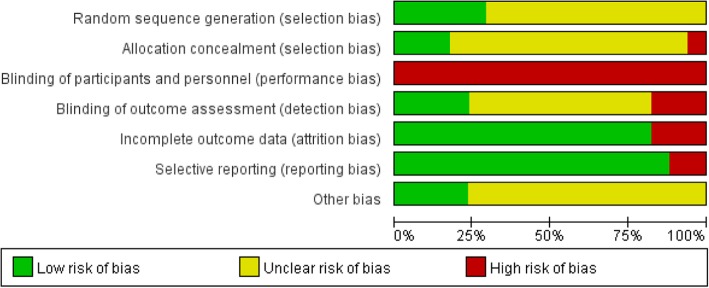
Risk of bias graph

**Fig. 3:**
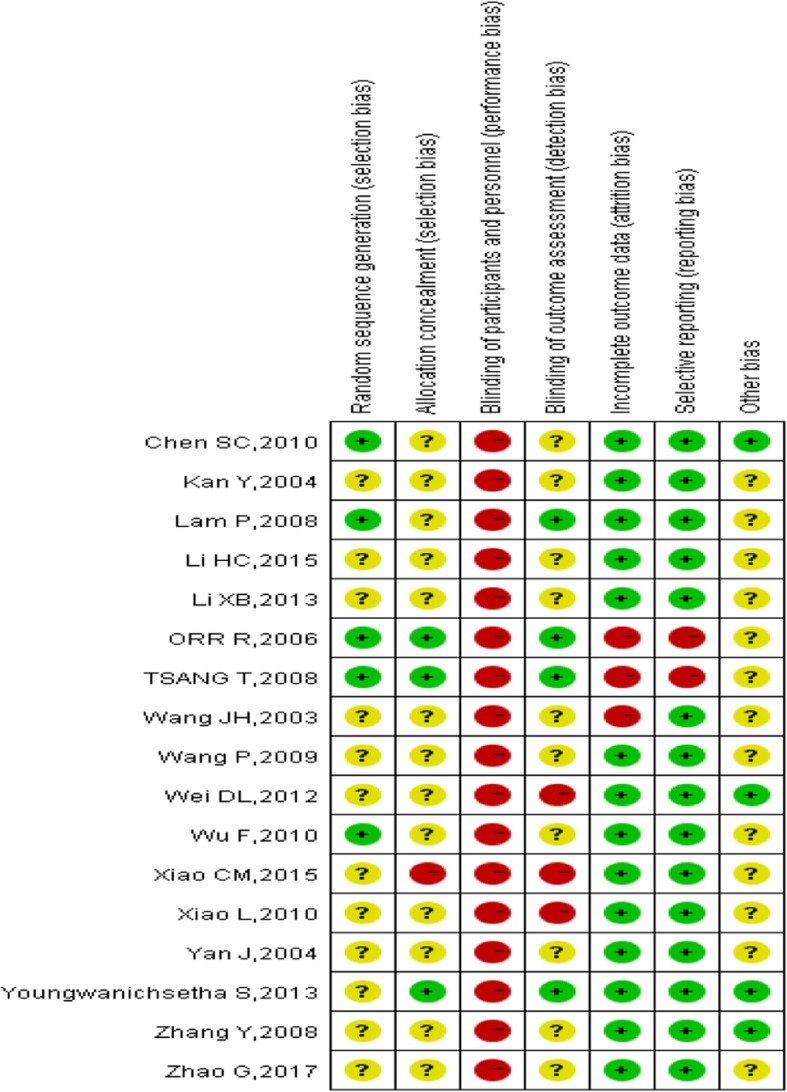
Risk of bias summary

The methodological quality of the included studies was generally low (Fig. 2 and Fig. 3). Because participants cannot be blinded to the Tai Chi intervention, performance bias could not be ruled out. Four studies [23–26] reported the random method of using a computer-generated random sequence, while one study [20] used only a random number table to divide participants into experimental and control groups. Twelve studies lacked descriptions of the method of random sequence generation. Fourteen studies did not describe how the allocation concealment was conducted, while three studies [25–27] used sealed opaque envelopes to perform the allocation concealment.

All studies lacked details on whether participants and administrators were blinded; however, it was clear that the blinding had been broken due to obvious differences between the intervention and control groups. Four trials used assessor blinding [24–27]. Fourteen studies reported all patient outcomes, while three studies [14, 25, 26] had a high drop-out rate; however, the drop-outs occurred at random. Additionally, intention-to-treat analyses were performed, one [14] of which provided detailed explanations. Two articles [25, 26] were from the same study and had different outcome indicators, but they had high selectivity bias and incomplete data. Most studies reported all outcomes listed in their methods section. The information necessary for judging the risk of other bias of all studies was insufficient.

### Meta-analysis of measured outcomes

#### FBG (fasting blood glucose)

**Fig. 4:**
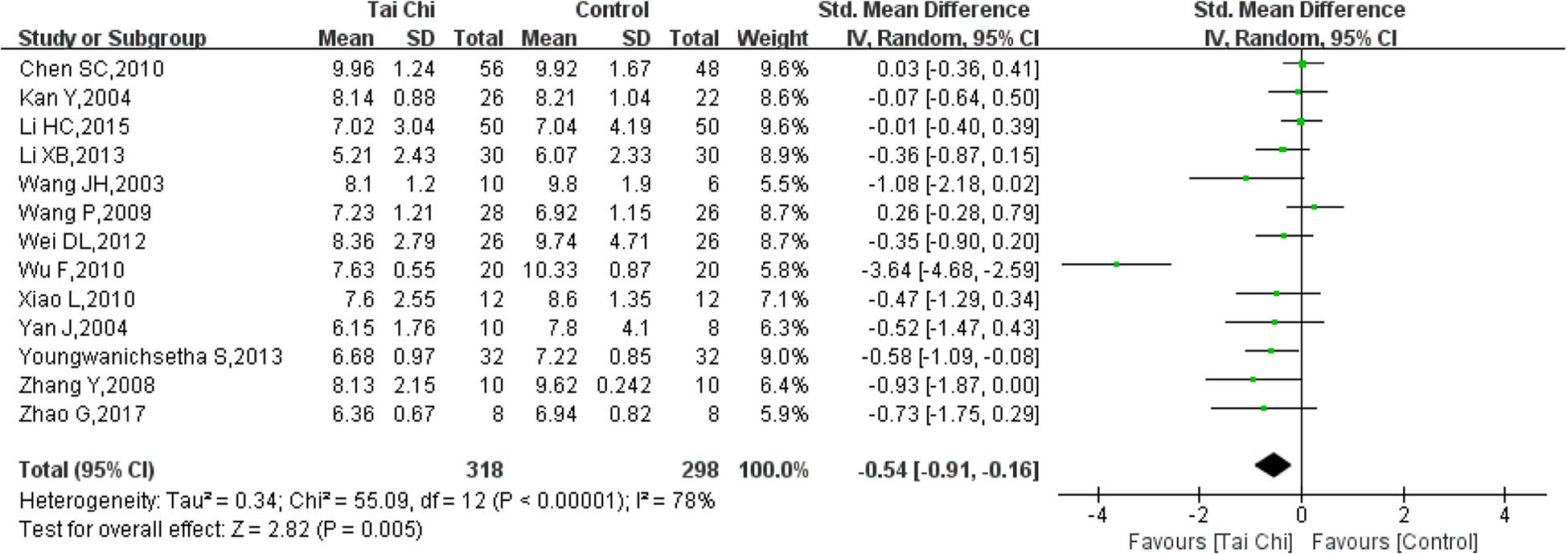
Forest plot of the comparison between Tai Chi and the control group for the outcome FBG

A total of 13 studies [11, 13–23, 27] with 616 patients were included, and 318 of the patients underwent a Tai Chi intervention. The heterogeneity was high (I^2^ = 78%, *P* < 0.00001), so we chose to conduct a quantitative synthesis using a random effects model. The combined result was statistically significant (SMD = − 0.54, 95% CI (− 0.91, − 0.16), *P* = 0.005) compared to the control group, showing favourable effects of Tai Chi on FBG (Fig. 4).

#### Subgroup analysis based on different training durations and styles of tai chi in the intervention groups

We first conducted subgroup analyses according to the different training styles of Tai Chi in the intervention groups. A total of 11 studies reported the training style of Tai Chi, while 2 studies did not report the Tai Chi style [11, 17]. Since 24 movements Tai Chi is a simplified version of Yang-style, we classified the two styles into one subgroup. The other styles, including Chen-style, Lin-style, Da Yuan Jiang Tang-style, and Tai Chi Ball, were in another subgroup. Then, the subgroup analysis was performed based on different intervention durations: ≤ 3 months and > 3 months.

**Fig. 5:**
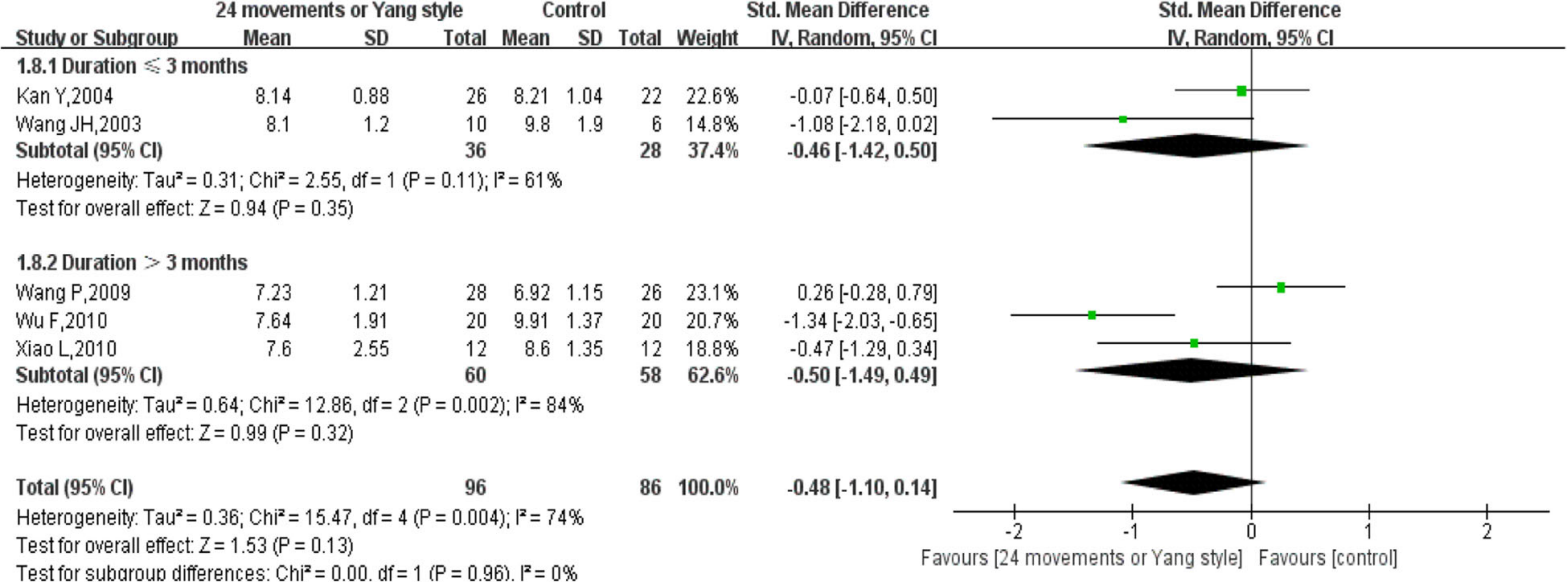
Forest plot of the comparison between 24 movements or Yang-style Tai Chi and the control group for the outcome FBG. The subgroup analysis was performed based on different intervention durations: ≤ 3 months and > 3 months

For the duration ≤3 months, the pooled results of 2 studies showed that 24 movements or Yang-style Tai Chi did not significantly reduce FBG (SMD = − 0.46, 95% CI (− 1.42, 0.50), *P* = 0.35), with high heterogeneity (I^2^ = 61%, *P* = 0.11) (Fig. 5). For the duration > 3 months, the pooled results of 3 studies also showed that 24 movements or Yang-style Tai Chi did not significantly reduce FBG (SMD = − 0.50, 95% CI (− 1.49, 0.49), *P* = 0.32), with high heterogeneity (I^2^ = 84%, *P* = 0.002) (Figs. 5, 13-15, 19, 21].

**Fig. 6:**
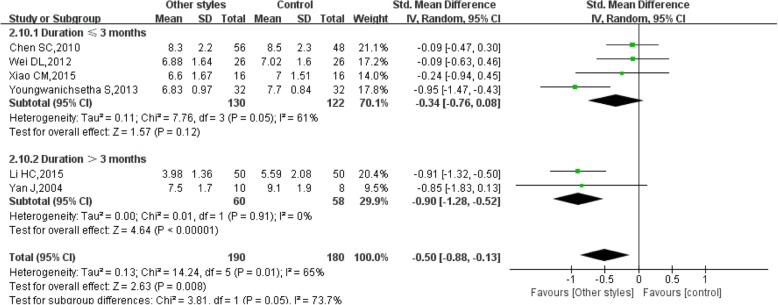
Forest plot of the comparison between other styles of Tai Chi and the control group for the outcome FBG. The subgroup analysis was performed based on different intervention durations: ≤ 3 months and > 3 months

For the duration ≤3 months, the pooled results of 4 studies showed that other styles of Tai Chi did not significantly reduce FBG (SMD = − 0.34, 95% CI (− 0.76, 0.08), *P* = 0.12), with high heterogeneity (I^2^ = 61%, *P* = 0.05) (Fig. 6). However, for the duration > 3 months, the pooled results of 2 studies showed that other styles of Tai Chi significantly reduced FBG (SMD = − 0.90, 95% CI (− 1.28, − 0.52), *P* < 0.00001), with low heterogeneity (I^2^ = 0%, *P* = 0.91) (Fig. 6) [16, 18, 21–23, 27].

### HbA1c (glycated haemoglobin)

**Fig. 7:**
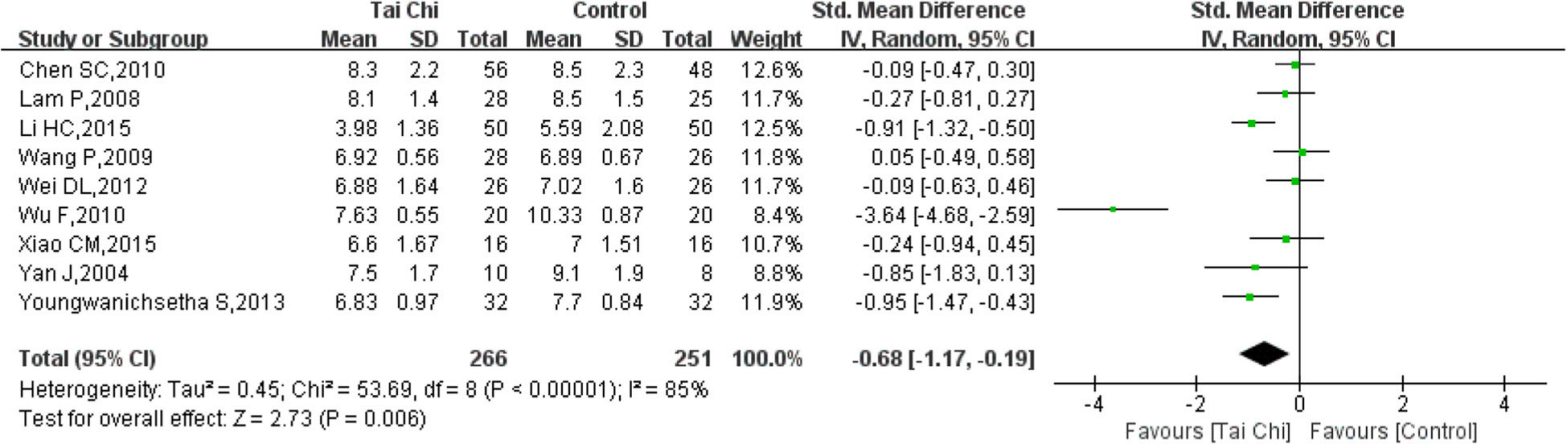
Forest plot of the comparison between Tai Chi and the control group for the outcome HbA1c

Nine studies [12, 15, 16, 18, 20, 21, 23, 24, 27] were included with a total of 517 patients, of whom 266 went through Tai Chi intervention. There was substantial heterogeneity among the included studies (I^2^ = 85%, *P* < 0.00001), so we first performed a quantitative synthesis using a random-effects model, which showed a significant difference between Tai Chi and the control group in reducing HbA1c (SMD = − 0.68, 95% CI (− 1.17, − 0.19), *P* = 0.006) (Fig. 7).

#### Subgroup analysis based on different training durations and styles of tai chi in the intervention groups

According to the different types of Tai Chi in the intervention groups, we conducted subgroup analyses. We also classified 24 movements and Yang-style Tai Chi into one subgroup. The other styles, including Chen-style, Lin-style, Da Yuan Jiang Tang-style, and Tai Chi Ball, were in another subgroup.

**Fig. 8:**
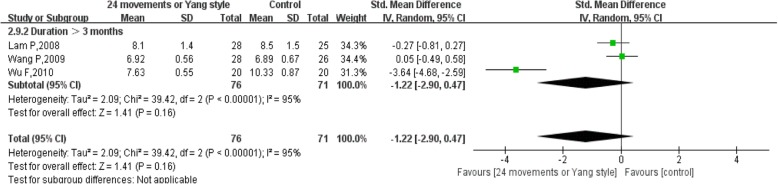
Forest plot of the comparison between 24 movements or Yang-style Tai Chi and the control group for the outcome HbA1c. The subgroup analysis was performed based on different intervention durations: ≤ 3 months and > 3 months

The pooled results of 3 studies comparing 24 movements or Yang-style and a control group showed no significant difference (SMD = − 1.22, 95% CI (− 2.90, 0.47), *P* = 0.16), with high heterogeneity (I^2^ = 95%, *P* < 0.00001) (Fig. 8). The durations of studies with 24 movements or Yang-style were all > 3 months [15, 20, 24].

**Fig. 9:**
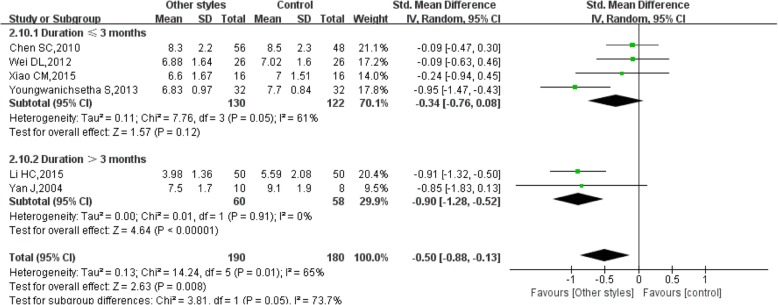
Forest plot of the comparison between other styles of Tai Chi and the control group for the outcome HbA1c. The subgroup analysis was performed based on different intervention durations: ≤ 3 months and > 3 months

The pooled results of 4 studies showed that other styles of Tai Chi did not significantly reduce HbA1c (SMD = − 0.34, 95% CI (− 0.76, 0.08), *P* = 0.12) for the duration ≤3 months, with high heterogeneity (I^2^ = 61%, *P* = 0.05) (Fig. 9). For the duration > 3 months, the pooled results of 2 studies showed that other styles of Tai Chi significantly reduced HbA1c (SMD = − 0.90, 95% CI (− 1.28, − 0.52), P < 0.00001), with low heterogeneity (I^2^ = 0%, *P* = 0.91) (Figs. 9, 12, 16, 18, 21, 23, 27].

### TC (total cholesterol)

**Fig. 10:**
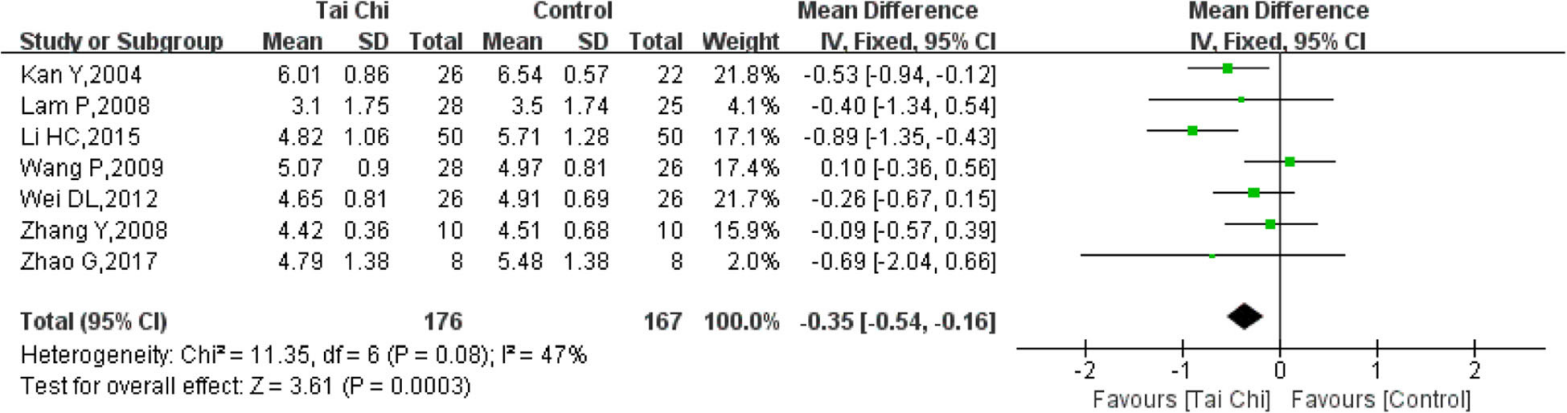
Forest plot of the comparison between Tai Chi and the control group for the outcome TC (total cholesterol)

Eight trials [11, 13–15, 18, 21, 22, 24] reported data on the change in TC following Tai Chi practice with 186 participants. The meta-analysis showed that there was significant heterogeneity among the studies (I^2^ = 94%, P < 0.00001). The analysis found that a small sample size was included in one study [14], and there was a large difference in subjects’ baseline values. A sensitivity analysis was used. Quantitative consolidation was performed after the study was removed. The heterogeneity of the seven studies was relatively low (I^2^ = 47%, *P* = 0.08). Pooled blood TC levels decreased in the Tai Chi group compared with the control group, and the difference was significant (SMD = − 0.35, 95% CI (− 0.54, − 0.16), *P* = 0.0003) (Fig. 10).

### TG (triglycerides)

**Fig. 11:**
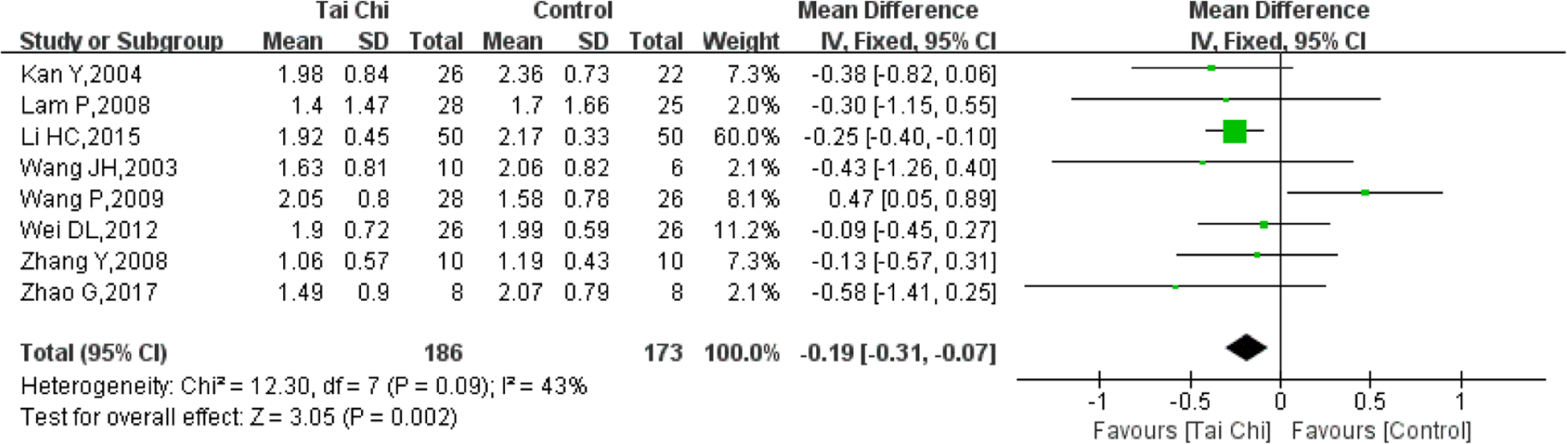
Forest plot of the comparison between Tai Chi and the control group for the outcome TG (triglycerides)

Eight studies [11, 13–15, 18, 21, 22, 24] reported TG levels in 359 patients, of whom 186 went through a Tai Chi intervention. The heterogeneity of the eight studies was relatively low (I^2^ = 43%, *P* = 0.09). The TG levels decreased in the Tai Chi group compared with the control group, and the difference was significant (SMD = − 0.19, 95% CI (− 0.31, − 0.07), *P* = 0.002) (Fig. 11).

### HDL-C (high-density lipoprotein cholesterol)

**Fig. 12:**
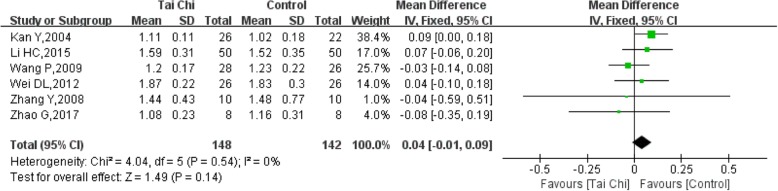
Forest plot of the comparison between Tai Chi and the control group for the outcome HDL-C (high-density lipoprotein cholesterol)

Six RCT trials [11, 13, 15, 18, 21, 22] with 290 participants provided data on blood HDL-C levels. The heterogeneity of the 6 studies was low (I^2^ = 0%, *P* = 0.54). The HDL-C levels improved in the Tai Chi group compared with the control group, but the difference was not significant (SMD = 0.04, 95% CI (− 0.01, 0.09), *P* = 0.14) (Fig. 12).

### LDL-C (low-density lipoprotein cholesterol)

**Fig. 13:**
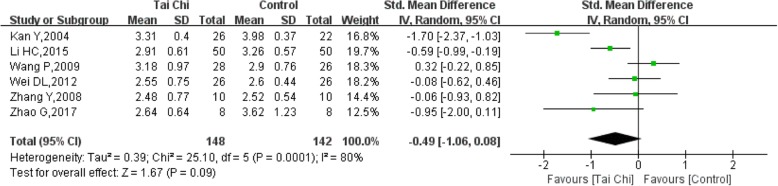
Forest plot of the comparison between Tai Chi and the control group for the outcome LDL-C (low-density lipoprotein cholesterol)

LDL-C levels were reported in 6 trials [11, 13, 15, 18, 21, 22] with a total of 290 participants. The heterogeneity of the 6 studies was high (I^2^ = 80%, *P* = 0.0001). The LDL-C levels decreased in the Tai Chi group compared with the control group, but the difference was not significant (SMD = − 0.49, 95% CI (− 1.06, 0.08), P = 0.09) (Fig. 13).

### BMI (body mass index)

**Fig. 14:**
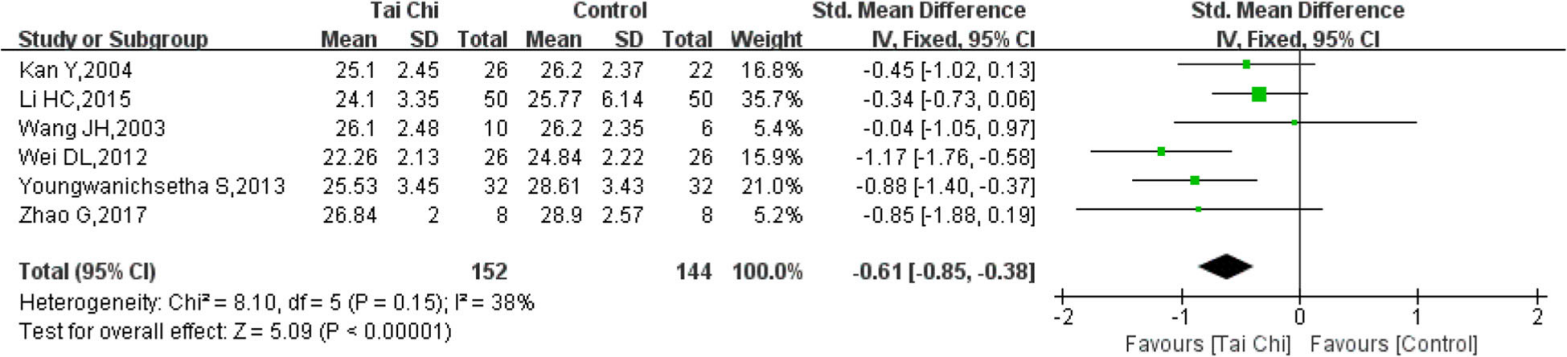
Forest plot of the comparison between Tai Chi and the control group for the outcome BMI (body mass index)

Six trials [13, 14, 18, 21, 22, 27] reported data on the change in BMI following a Tai Chi practice in 296 participants. The heterogeneity of the 6 studies was relatively low (I^2^ = 38%, *P* = 0.15). The BMI decreased in the Tai Chi group compared with the control group, and the difference was significant (SMD = − 0.61, 95% CI (− 0.85, − 0.38), *P* < 0.00001) (Fig. 14).

Safety monitoring: Twelve studies did not report adverse events. Five studies [11, 23, 25–27] reported that there were no adverse events.

**Fig. 15:**
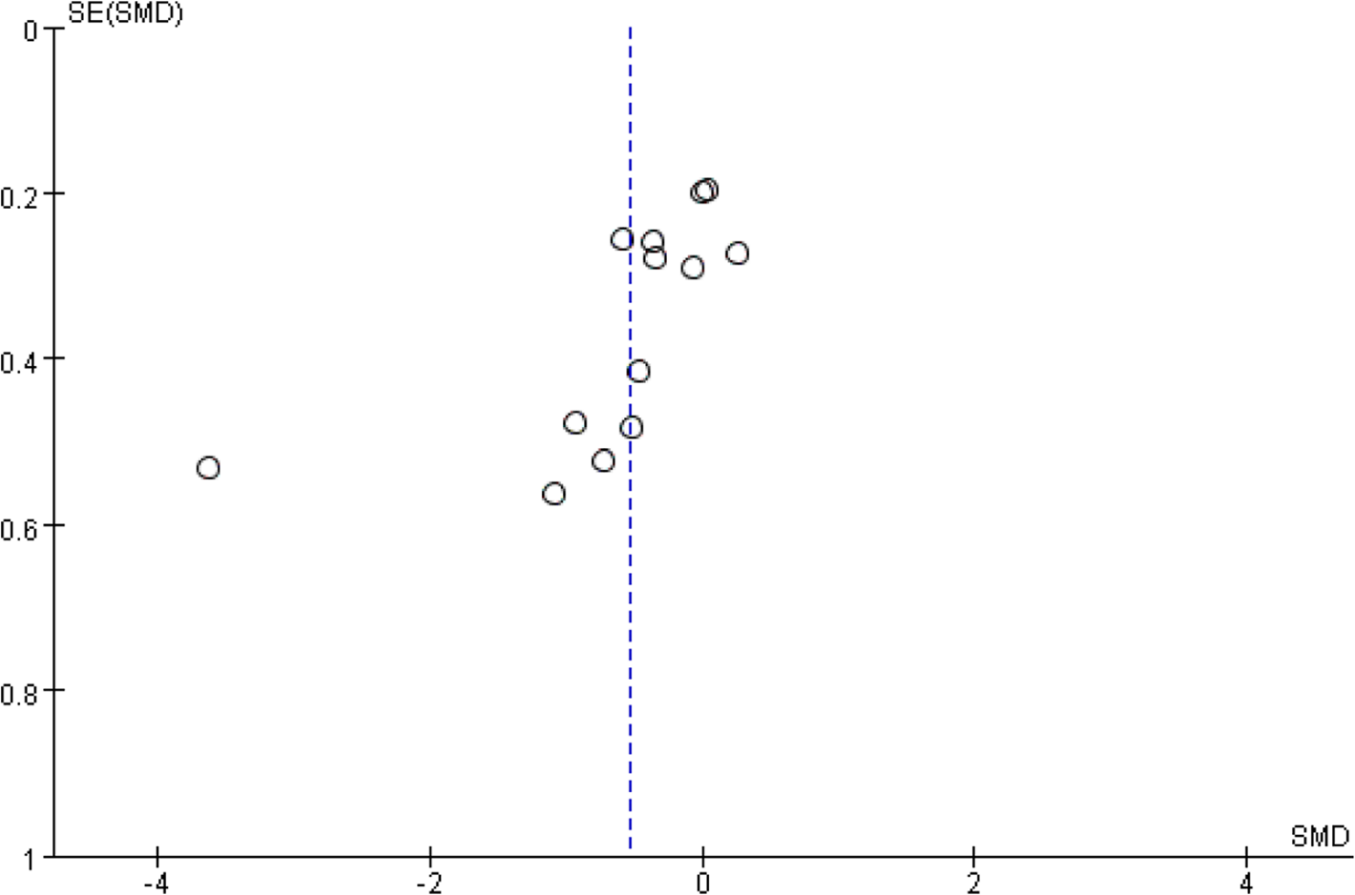
Evaluation of publication bias for FBG

Publication bias: The FBG included in the study was selected as the indicator. It can be seen that the graph is obviously asymmetrical (Fig. 15). There might have been publication bias in the comparison of Tai Chi and the control group, as the results showed that the distribution of the included studies on both sides of the funnel plot was asymmetric.

## Discussion

Tai Chi has been widely practised since ancient times. In recent years, it has gained increased popularity in health care, especially among seniors. Tai Chi has been proven to be a low-to-moderate-intensity aerobic exercise [28–31]. Previous studies have revealed that certain physical activities, such as Tai Chi, can reduce insulin resistance [32], reduce the function of blood coagulation factors, and promote blood circulation [33]. But the conclusions of previous systematic reviews were not uniform. Most of them, except the latest SR, have suggested that the evidence is insufficient to support Tai Chi as an effective therapy for type 2 diabetes. Meanwhile, the effects caused by different training durations and styles of Tai Chi have not been evaluated. By using a quantitative synthesis, our review showed that Tai Chi seems to be effective for blood glucose control in patients with T2DM, and first showed that different training durations and styles could result in variable effectiveness.

### Summary of Main results

Overall, the included studies proved that Tai Chi might be beneficial for treating T2DM. This systematic review and meta-analysis showed that blood glucose control was greater among Tai Chi intervention groups than in control groups. Significant differences were found in reducing FBG and HbA1c, although the heterogeneity was high.

Considering the subgroup analysis for FBG, the pooled results showed that 24 movements or Yang-style Tai Chi did not significantly reduce FBG, either after a duration ≤3 months or a duration > 3 months [13–15, 19, 21]. However, the pooled results showed that other styles of Tai Chi did not significantly reduce FBG after a duration ≤3 months with high heterogeneity, while a significant difference was found after a duration > 3 months with low heterogeneity [16, 18, 21–23, 27]. There was no significant difference in reducing HbA1c between 24 movements or Yang-style and the control group, and the duration in all studies was > 3 months. However, other styles of Tai Chi significantly reduced HbA1c after a duration > 3 months with low heterogeneity, while a significant difference was not found after a duration ≤3 months with high heterogeneity [12, 16, 18, 21, 23, 27].

The exercise intensity of Tai Chi mainly depends on the training style, posture, frequency and duration. Furthermore, it is a complex, multi-component intervention that integrates physical, psycho-social, emotional, spiritual, and behavioural elements, and not just the utilization of oxygen [34, 35]. Our review first showed that different training durations and styles could result in variable effectiveness. The subgroup analysis was conducted according to the training durations and styles of Tai Chi. The results showed that other styles of Tai Chi significantly reduced FBG and HbA1c after a duration > 3 months. However, 24 movements or Yang-style Tai Chi had no effectiveness in reducing FBG or HbA1c, either after a duration ≤3 months or > 3 months. Our study also focused on TC, TG, HDL-C, and LDL-C levels, and we found that Tai Chi has a beneficial effect on controlling blood lipid levels.

In recent years, more and more research has focused on the Tai Chi training style, posture, frequency and duration. Studies have shown that Tai Chi might affect diabetic patients by improving the motor nerve conduction velocity of the bilateral central and sacral nerves, improving the distal sensory latency of the bilateral ulnar nerves, and increasing vascular function, especially increasing the vascular reactivity index [36, 37]. The results of one study showed that the longer the Tai Chi practice, the stronger the tactile space acumen [38]. Some studies have shown that Tai Chi can enhance the use of the active neural networks through an all-inclusive mind-body approach [39, 40]. Not only has it been shown that the effects of Tai Chi on proprioception in the ankle and knee joints were better than in sedentary controls, but also that the effects on ankle kinaesthesis were better than in swimmers and runners [41]. Such training might decrease the potential for developing joint injuries or foot ulcers, which occur in a large proportion of people with PN [41]. It can be seen that Tai Chi can play an important role in such combinatorial therapies.

The quality of the included studies was not high, with twelve studies lacking a description of the randomization method, and fourteen studies not mentioning allocation concealment. In addition, selection bias existed. The outcome assessors were successfully blinded in four studies [25–27], while no studies successfully blinded the researchers or participants. Regarding the safety of Tai Chi, twelve studies didn’t reported any adverse events due to Tai Chi. Five studies [11, 23, 25–27] reported that there were no adverse events. So the evidence was insufficient. More research on the safety assessment of Tai Chi exercise for people with type 2 diabetes is needed.

### Limitations

The greatest concern with this study is regarding the distribution of the included studies in the meta-analysis. Most studies were conducted in Asian countries such as China (Taiwan) or Australia; no studies were from the USA or western countries. We only performed a search for Chinese and English studies, and it is possible that articles may have been published in other languages, which caused the publication bias. Moreover, in our review, the styles of Tai Chi intervention and outcome measurement tools varied. We could not make higher quality and more comprehensive subgroups due to the limited number of included studies. This may have influenced the explanatory effect and the soundness of the pooled effects. The methodological quality of the included trials was not promising; in addition to the fact that Tai Chi is hard to be blinded to, the included studies have other flaws, such as poor randomization. Undoubtedly, the credibility of subgroup effects in this study was low, due to the poor quality of the studies included in this meta-analysis. At the same time, according to the criteria to assess the credibility of subgroup effects [42–44], the credibility of subgroup effects in this study was still low. Although we have determined the characteristics of the study in advance and tried to select a small number of research features, the reliability of the subgroup effect is not guaranteed. This study was the first discussion of different training durations and styles. The results of the subgroup analysis should be treated with caution.

Heterogeneity among the studies was significant, which may be explained by the inconsistent measurement of exercise intensity. We conducted a sensitivity analysis of the included RCTs and found two studies that could have been the main source of heterogeneity. The target heart rate was used as a measurement of exercise intensity in both studies, which had a great impact on the combined results. The intervention in the control group, which included standard diabetes care, active control, and no treatment, was an important factor in generating heterogeneity. Subject inclusion criteria was also a non-negligible factor in generating heterogeneity. Although the inclusion criteria for the study uniformly included without serious complications, there were some differences in the detailed criteria for each study. For example, some studies have defined blood glucose, blood pressure, and blood lipid levels in the criteria. The variation in training styles, durations and outcome measurement tools resulted in substantial differences in exercise intensity.

### Implications for research

We summarized the current condition of Tai Chi for type 2 diabetes and provided information to support a future clinical trial. Although this study showed that Tai Chi might be effective for controlling blood glucose and blood lipid levels, the current evidence and potential findings should be interpreted carefully because of the poor methodological quality of these studies, insufficient evidence for safety and clinical heterogeneity. Future studies should pay more attention to the effects of the Tai Chi styles and exercise intensity measurement methods on patients with type 2 diabetes. Overall, Tai Chi exercise may be used to control blood glucose and blood lipid levels.

## Conclusions

Tai Chi seems to have effectiveness in treating type 2 diabetes compared to control interventions. Different training durations and styles result in variations in effectiveness. However, the evidence was insufficient to support whether long-term Tai Chi training is more effective. Due to the poor quality of the methodology, more high quality randomized controlled studies should be conducted to substantiate the effects of Tai Chi on controlling blood glucose and blood lipid levels. In addition to investigating the training frequency/duration of Tai Chi in regard to maximizing health benefits, particularly the exercise intensity measurement methods, researchers should also investigate the effects of various styles of Tai Chi on health-related parameters.
